# Venom Ophthalmia and Ocular Complications Caused by Snake Venom

**DOI:** 10.3390/toxins12090576

**Published:** 2020-09-08

**Authors:** Kun-Che Chang, Yu-Kai Huang, Yen-Wen Chen, Min-Hui Chen, Anthony T. Tu, Yen-Chia Chen

**Affiliations:** 1 Spencer Center for Vision Research, Byers Eye Institute, School of Medicine, Stanford University, Palo Alto, CA 94304, USA; kunche@stanford.edu; 2 Department of Ophthalmology, Louis J. Fox Center for Vision Restoration, University of Pittsburgh School of Medicine, Pittsburgh, PA 15213, USA; 3 Graduate Institute of Clinical Medicine, College of Medicine, Kaohsiung Medical University, Kaohsiung 80708, Taiwan; yukaih@gmail.com; 4 Division of Neurosurgery, Department of Surgery, Kaohsiung Medical University Hospital, Kaohsiung 80708, Taiwan; 5 Department of Surgery, Kaohsiung Municipal Ta-Tung Hospital, Kaohsiung 80145, Taiwan; 6 Department of Chest Medicine, Taipei Veterans General Hospital, Taipei 11217, Taiwan; albert6369@gmail.com; 7 Institute of Clinical Medicine, National Yang-Ming University, Taipei 11221, Taiwan; 8 Enkang Clinic, New Taipei 23144, Taiwan; chen.minhui@gmail.com; 9 Department of Biochemistry and Molecular Biology, Colorado State University, Ft. Collins, CO 80523, USA; atucsu@gmail.com; 10 Emergency Department, Taipei Veterans General Hospital, Taipei 11217, Taiwan; 11 Department of Emergency Medicine, School of Medicine, National Yang-Ming University, Taipei 11221, Taiwan; 12 National Defense Medical Center, Taipei 11490, Taiwan

**Keywords:** snake venom, snakebite, spitting venom, nuchal gland, corneal edema, retinal hemorrhage

## Abstract

Little is known about the detailed clinical description, pathophysiology, and efficacy of treatments for ocular envenoming (venom ophthalmia) caused by venom of the spitting elapid and other snakes, as well as ocular complications caused by snake venom injection. In this paper, we review clinical information of case reports regarding venom ophthalmia and snake venom injection with associated ocular injuries in Asia, Africa, and the United States. We also review the literature of snake venom such as their compositions, properties, and toxic effects. Based on the available clinical information and animal studies, we further discuss possible mechanisms of venom ophthalmia derived from two different routes (Duvernoy’s gland in the mouth and nuchal gland in the dorsal neck) and the pathophysiology of snake venom injection induced ocular complications, including corneal edema, corneal erosion, cataract, ocular inflammation, retinal hemorrhage, acute angle closure glaucoma, as well as ptosis, diplopia, and photophobia. Finally, we discuss the appropriate first aid and novel strategies for treating venom ophthalmia and snake envenoming.

## 1. Introduction

**Figure 1:**
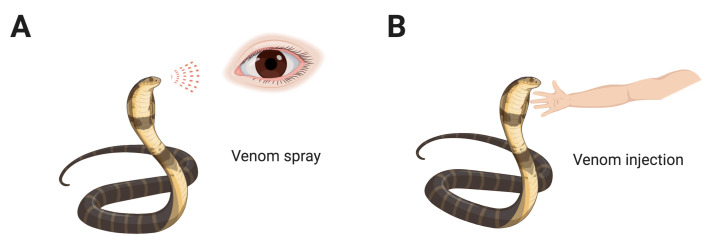
Snake venom ophthalmia via venom spray (**A**) and venom injection (**B**).

**Figure 2:**
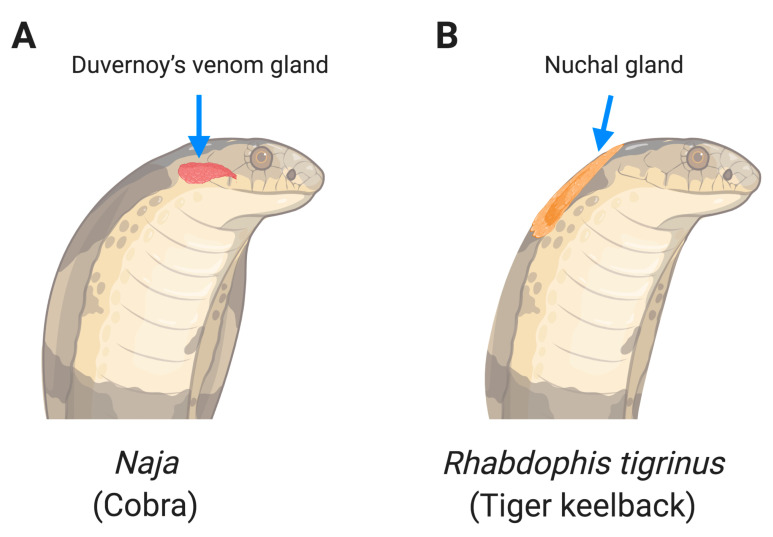
Duvernoy’s gland (**A**) and nuchal gland (**B**) are two different systems of venom spray.

Snakes are carnivorous reptiles, which are cold-blooded animals. They inhabit every continent and ocean (sea snake) [1] except Antarctica. Some snakes are venomous while some are not. Venomous snakes comprise four families, including *Colubridae*, *Elapidae*, *Viperidae*, and *Atractaspididae* [2]. In this review, we distinguish between venom-induced ophthalmias, venom spray (Figure 1A), and venom injection (Figure 1B). We further discuss two sources of venom spray, initiating from the modified salivary gland (Duvernoy’s glands, Figure 2A) or prey-derived toxin storage glands (nuchal gland, Figure 2B). Among snake species, spitting cobras, a member of the *Elapidae* family snakes, can spit venom from Duvernoy’s gland while irritated or threatened. The tiger keelback, a member of the *Colubridae* family snakes often found in East Asia, conducts a toxin spray from its nuchal gland. After a toxin spray attack, the eyes are most often affected, causing inflammatory responses in the anterior segment of the eye. In venom spray ophthalmia, many symptoms such as hyperemia, uveitis, and corneal erosions are common complications after venom spray [3]. 

Ocular complications in the posterior segment are often observed after snakebite. An epidemiological study of 180 snakebite patients in India reported that 69% of victims present with ocular neuroparalytic manifestations [4]. Most viperide snakes discharge venom as the fangs hook into the victim, and then immediately release the bite. However, some species (e.g., *Lachesis*) may hold the bite, discharging a larger amount of venom to kill the victim, especially in times of starvation. So far, about 59 protein families have been identified in venoms, which are capable of inflicting neurotoxicity, cytotoxicity, hemotoxicity, and myotoxicity, [5] and most snake venoms have mixed effects. Hemotoxins in snake venom cause hemolysis, destruction of erythrocytes, and blood clotting. Since hemotoxins are abundant in *viperidae*, ocular hemorrhage and secondary inflammatory responses are the most common ocular complications by viper snake envenoming. Neurotoxins in snake venom cause neurological disorders in the eye, such as ocular muscle paralysis, ptosis, and diplopia. Without immediate treatment, patients can be left with permanent tissue damage, blindness, or even death from respiratory muscle paralysis. In this review, we focus on venomous neurotoxins and hemotoxins and the possible ocular pathophysiology after venom spray or venom injection. We also discuss the current development of therapeutic strategies for treating venom-induced ocular complications.

## 2. Snake Venom/Toxin Cause Ocular Complications

### 2.1. Spitting Venom and Sprayed Toxin

#### 2.1.1. Venom Spat from Duvernoy’s Gland

*Naja* is a genus of venomous elapidae, also known as cobras. Several species of *Naja* cobras can spit their venom from Duvernoy’s gland (Figure 2A), where venom is generated and stored, through fangs onto victims. The cobras can spit venom onto targets about 1.5 m away [6]. To understand the cobras’ spitting behavior, a study utilized photos of human faces or hands to trigger spitting of two cobras, *Naja nigricollis* and *N. pallida* [7]. That study revealed that most cobras only respond to moving faces but not to hands within 10 s (79% of *N. nigricollis* and 67% of *N. pallida*). Spitting patterns on the photos showed that the venom streamlines are either between the eyes or at one eye, suggesting the intent to hit at least one eye of an aggressor. Another group investigated target tracking during cobra venom spitting [8]. They observed that cobras perform rapid cephalic oscillations to coordinate the target’s movements, for increasing the chance of hitting the eyes. Due to high occurrences of venom spitting on the eye, we review cases of venom-spit ophthalmia by cobras in Asia [3,9,10], Africa [3], and United States [11] as well as by rattlesnakes in the United States [12,13,14] (Table 1).

##### In Asia

*N. atra*, also called the Chinese cobra, is found most in Taiwan and China. An epidemiological survey of venom-spit ophthalmia from 1990 to 2016 in Taiwan studied a total of 39 cases suffering from *N. atra* [10]. Data showed that most cases involved a single eye (82%) to male (95%) adults between 18 to 59 years old (90%). About half of the cases occur during catching (51%), while most occurred in hot seasons (spring and summer, 92%). About nine in ten have ocular symptoms including ocular pain (90%) and redness (85%), conjunctivitis (67%), and corneal injury (59%). After immediate water irrigations, most cases (77%) were symptom free after the acute stage. Although no children were reported in Taiwan, snake venom spitting was also considered one of the causes of eye burn in children in other courtiers [15]. Another venom-spit ophthalmia by *N. atra* was reported from an 83-year-old woman in Hong Kong while she was trying to kill the cobra [9]. After being treated with 0.5% chloramphenicol and 0.12% prednisolone eye drops, she went home without severe symptoms.

*N. siamensis*, also called the Indochinese spitting cobra, is a species most found in Southeast Asia. A 45-year-old man in Laos was spat on by *N. siamensis* on both eyes, about 2 m away from the cobra [3]. Luckily, he presented without evidence of corneal injury. He received topical epinephrine drops 1:10,000 and recovered completely in 24 h.

*N. naja*, also called the Indian cobra, is a species found in India and neighboring countries. A male cobra player was spat on by *N. naja* on his left eye [3]. Since he was sprayed at from a short distance (20 cm), he experienced eye pain, conjunctival inflammation, and swelling of the eyelids, despite an immediate tap water wash. After treatment, his symptom was alleviated.

##### In Africa

*N. pallida*, also called red spitting cobra, is a native spiting cobra in Africa. Two cases were reported; one is the graduate student who got venom spray during snake transfer and another one got attacked while photographing a juvenile specimen. They both felt pain in their eyes but recovered after treatment without sequelae [3].

*N. nigricollis*, also called the black-necked spitting cobra, is found mostly in sub-Saharan Africa. There were six case reports in Nigeria [3,16,17,18]. Two victims had corneal edema, hypopyon eye, and uveitis [16,18], while two victims suffered permanent vision loss [18]. Most cases of attacks/by *N. nigricollis* took longer to recover than ones by *N. pallida*, suggesting that venom from *N. nigricollis* might be more toxic to the eye than *N. pallida*.

*Hemachatus haemachatus*, also called the ringhal or ring-necked spitting cobra, is a species active in parts of southern Africa [3], which is not an elapidae that commonly uses venom-spit to trap the prey. A young male herpetologist got venom spat into his right eye from a distance of about 1 m while photographing a captive specimen. After flushing the eye with tap water, his eye was slightly pink but otherwise normal the next morning.

##### In United States

Although cobras are not native in the United States, two cases of venom-spit ophthalmia by imported African cobras were reported in Southern California [11]. The first patient was a 30-year-old male snake handler that was sent to the UCLA emergency department 2 h after his left eye was exposed to venom from *N. nigricollis*. After treatment with otic drops (ciprofloxacin hydrochloride four times daily) and a cycloplegic drop (cyclopentolate three times daily) for the left eye, his visual acuity of the left eye improved from 20/50 to 20/30 at one month after injury. He went home with a mild long-term decrease in visual acuity of the affected eye. Another patient was a 22-year-old man that was also sent to UCLA emergency department two days after his left eye was exposed to venom from an African spitting cobra. Slit-lamp examination was remarkable for a large epithelial defect of the left cornea. He was then treated by topical steroid drops (prednisolone acetate 1% four times daily) for one week. By two weeks after injury, visual acuity returned from 20/30 to 20/20 in the left eye.

*Crotalus atrox,* commonly called rattlesnake, belongs to the viperidae family and is native to America. Similar to the spitting cobra of Asia, rattlesnake is the most common cause of venom-spit ophthalmia in the United States. Three cases of crotalid venom-spit ophthalmia are reviewed here [12,13,14]. One woman was spat on by a rattlesnake on both eyes while trying to beat the snake with a hammer. She was then sent to the emergency department and reported significant pain, photophobia, and foreign body sensation. She received antivenom and tetracaine ophthalmic drops (0.5%) for ophthalmic treatment and intravenous morphine sulfate for reducing pain [14]. Upon follow-up examination, her visual acuity was preserved. The other two women immediately irrigated their eyes with tap water after venom exposure and reported relief afterward. The follow-up diagnoses were not successful for both cases [12,13].

#### 2.1.2. Toxin Sprayed from Nuchal Gland

In addition to having Duvernoy’s gland to produce venom, some snakes also have another organ, the nuchal gland, to store venomous fluid (Figure 2B). The nuchal gland, also named nucho-dorsal gland or cervical gland, is a special defensive system in some species of snakes such as the *Rhabdophis* genus [19,20]. These snakes consume a poisonous prey and conserve the poison in their nuchal glands to spray the stored poison for defense. A first observation of snakes with nuchal glands was reported in 1935 by Nakamura [21] and nine more species were reported by Smith three years later [22]. Although most nuchal glands were found at the back of the neck, some nuchal glands were observed across the whole body on some snakes [19]. To date, there are 13 species of snakes found in three genera (*Rhabdophis*, *Macropisthodon*, and *Balanophis*) that have nuchal glands [19]. They consume toads (Bufonidae) and store the toad’s poison in their nuchal glands [23]. Here, we review the cases of venom ophthalmia by Rhabdophis tigrinus via their nuchal glands in Japan and Taiwan.

##### In Japan

Similar to spitting cobras, *R.t. tigrinus* can spray nuchal gland-derived fluid over a distance of over one meter [24]. In the past 30 years, three cases of fluid-sprayed ophthalmia by *R.t. tigrinus* were reported in Japan [24,25,26]. Their ophthalmologic examination revealed conjunctivitis, keratitis, and corneal clouding. After short clinical courses, their ocular complications recovered with good prognosis. Other than proteolytic enzymes in venom, the bufadienolides in the nuchal gland secretion of *R.t. tigrinus* are considered the crucial component in causing human ophthalmia [24,25,26].

##### In Taiwan

*R.t. formosanus* (Taiwan tiger keelback) is a conserved animal in Taiwan. There is a case of a 40-year-old man whose right eye was sprayed on by the *R.t. formosanus* [27]. This is the first known case of eye injury caused by the nuchal gland secretion of *R.t. formosanus* in Taiwan. The patient’s clinical symptoms included foreign body sensation, progressive burning pain, and blurred vision. Diffuse superficial punctate keratitis, corneal stromal edema, and conjunctival congestion were observed by ophthalmic examination. After treatment with a topical corticosteroid, antihistamine, and antibiotic, he recovered well without any sequela.

### 2.2. Snake Venom Injection

Other than venom spitting/toxin sprayed, snakebite (snake venom injection) is another common cause of ocular complications (Table 2). Most snakebites occur on limbs and venomous toxins circulate in the host body, reaching the eye and leading to ocular complications. Since retina and choroid are rich in vessels [28], posterior segment complications are often observed in snakebite victims. Common symptoms are central retinal artery occlusion (CRAO) [29,30,31,32], retinal or vitreous hemorrhage [33], and macular infarction [33]. A rare case even reported that a 13-year-old boy was diagnosed with retinal detachment after snakebite [34]. Even after receiving immediate treatment for posterior complications, most patients claim their vision is not the same as it was. Since the cornea and lens are avascular tissues, snake venoms do not directly injure these tissues. However, some anterior segment complications such as corneal striae, cataract, anterior pseudohypopyon, anterior ischemia, and iris atrophy were also observed in the eye of snakebite victims [35,36]. These complications in the anterior segment might be attributed to the secondary inflammatory effects in the posterior segment. Since corneal injury is not a common complication of snakebites, a clinical study even reported the safety and suitability of using donor corneas from snakebite victims for corneal transplantation [37].

In addition to damage of ocular tissues, acute angle-closure glaucoma (AACG) following snake venom injection was also reported [38,39], in which the anterior chamber fluid circulation is inhibited in the eye leading to intraocular pressure (IOP) increase. A statistical study from India reported that about 50% of snakebite victims developed bilateral AACG one year after the incident [40]. One patient even developed optic neuropathy one month after AACG diagnosis [39]. Due to different characteristics of venoms, some patients display ocular muscle paralysis [41,42], ptosis, and diplopia [43].

Snake venom injection may cause a lifelong ocular morbidity. A case in Nigeria showed a 10-year-old boy bitten by a brownish snake (presumably carpet viper). He was brought to the Emergency Pediatric two weeks after snakebite with local swelling, epistaxis, bilateral proptosis, and exposure keratopathy. Although the antivenom was administered to save his life, he left the hospital with bilateral blindness [44]. Another case reported a 14-year-old Indian boy who was given IV injection of methylpredinisolone in the hospital after snakebite [45]. He was diagnosed with vitreous hemorrhage in his right eye. At follow-up after one month, he still had no perception of light in his right eye due to no improvement in the vitreous hemorrhage. Therefore, he lost vision in one eye.

Apart from bites to the limbs, only a few cases have been reported in which the eye was bitten directly. A 34-year-old man who suffered an ocular bite by a venomous snake, *Agkistrodon acutus* (hundred-pace snake, one of the most toxic snakes in the world) to his right eye causing subconjunctival hemorrhage, sever necrosis of the corneal endothelium, and exophthalmos [46]. Although he received immediate ocular surgery, maintaining his vision and preventing infection was a challenge for him. Another 5-year-old female patient suffered from snake venom injection directly to her eye, leading to eye enucleation due to sever ocular necrosis [47]. However, another victim was bitten by *Python molurus* (non-venomous) to his left eye, causing corneo-scleral laceration and hyphaema [48]. After appropriate treatments, including Argon laser retinopexy, he was discharged after one week. Luckily, he recovered very well, in which his wound had healed well and the injured retina remained attached at six weeks follow-up. Based on the three cases above, the toxic effects of venom may determine the extent of visual damage following snakebite to the eye directly.

### 2.3. Venom Exposure by Accidental Touch

*Notechis scutatus*, also called the tiger snake, is found in south Australia. Tiger snakes do not spit venom onto their victims. However, there is a case of venom-exposed ophthalmia due to carelessly touching one’s eye while handling the tiger snake’s venom sample [3]. This is a rare case of venom-induced ophthalmia from a non-spitting snake.

## 3. Pathophysiology of Venom Induced Ocular Complications

### 3.1. Mechanism of Spitting-Venom/Sprayed-Toxin Induced Ocular Complications

The cornea is the transparent part of the eye that covers most of the anterior segment. Corneal injury often causes blurred vision even vision loss. In venom-spit ophthalmia, the cornea is the first tissue exposed to venom. Corneal edema, conjunctival inflammation, and uveitis are the common diagnoses of venom-spit ophthalmia. These symptoms result from the intrinsic release of histamine and acetylcholine triggered by venom enzymatic components such as collagenase and protease [49]. Due to the neurotoxins in venom, pupillary constriction dysfunction leading to photophobia has also been reported by spitting cobra venom attacks. Other than direct neurotoxic effects, corneal injury and uveitis may also indirectly cause photophobia.

To better understand the mechanisms of spitting-venom on corneal injury, several studies used rabbit eyes as a model. Cobra (*N. nigricollis*) venom was shown to penetrate the corneal epithelium and bind to the corneal stroma. Corneal complications occurred 30 min after initiation and reached the maximum severity at 12 h [49], displaying nitrogen mustard-like effects (similar to chemical burn) on rabbit corneas [50]. To compare the effects of venom from different snake species, a group conducted an ex vivo eye irritation test (EVEIT) [51] and found that venoms from spitting elapids Naja (*N. mossambica, N. nigricollis*) but not non-spitting vipers (*Bothrops jararaca* and *B. lanceolatus*) cause increased thickness of rabbit corneas [51]. Interestingly, the venom from *N. naja* also causes the corneal symptom, which can be alleviated after a 10 min water rinse, suggesting that the venom from *N. naja* has less tissue penetrating ability than the other two *Naja* species. Among these experimental studies, it seems that spitting venoms of African cobras (*N. nigricollis* and *N. mossambica*) cause stronger corneal damage than the one from Asia (*N. naja*). In addition, there is no evidence showing that venom-spit onto the eye can enter circulation, explaining why there were no systemic manifestations or mortality report by venom-spit ophthalmia.

In contrast to venom in the Duvernoy’s gland, the toxin in the nuchal gland is mainly derived from prey, such as toads. The sprayed toxin from *R. tigrinus* (tiger keelback) mainly causes corneal and conjunctival complications similar to symptoms caused by spitting venom from cobras. Bufadienolides, characterized as digitalis-like compounds (DLCs), are found in toad poison and conserved in the nuchal glands of the tiger keelback [52]. The sodium-potassium adenosine triphosphatase (Na^+^/K^+^ ATPase) pump is known to maintain corneal transparency and aqueous humor secretion in the corneal endothelium [53]. The inhibitory property of DLCs on Na^+^/K^+^ ATPase might explain how bufadienolides from nuchal gland secretions cause corneal edema. A group in Japan purified bufadienolides from the nuchal gland secretion of *R. tigrinus* and further confirmed that bufadienolides can cause iritis, conjunctivitis, and keratitis in rabbit eyes [54].

### 3.2. Mechanism of Venom Injection Induced Ocular Complications

Three main enzymatic proteins have been discovered in viper venom, including proteases (serine and metalloproteinases), oxidases (L-amino acid oxidases, LAAOs), and phospholipases (especially phospholipases A_2_, PLA_2_) [55]. Hemotoxins in venom are one of the key components that cause life-threating complications, such as systemic bleeding, coagulopathy, thrombocytopenia, and hemolysis [56,57]. In the eye, the retina and choroid are blood vessel-rich tissues. Previous cases reported that hemotoxin-induced retinal hemorrhage is the main cause of permanent vision loss [44,45]. Possible explanations for damage despite treatment may be the delay in administering antivenom or the insufficient neutralizing capacity of antivenom. However, the viper envenoming caused retinal/vitreous hemorrhage will be recovered better if appropriate antivenom is administered timely [40]. Among hemotoxins, snake venom metalloproteinases (SVMPs) have been considered to cause hemorrhage by proteolytic destruction of basement membrane and extracellular matrix surrounding capillaries and small vessels [55,58,59,60]. This was studied in mouse and rabbit skin models [61,62], in which intradermal injection of SVMPs leads to dermal hemorrhage. These studies may infer the effect of SVMPs on retinal hemorrhage. In addition to SVMPs, LAAOs were also found to cause hemorrhage in a mouse skin model [63]. SVMPs and PLA_2_ also inhibit human and mouse platelet aggregation in vitro [59,64,65,66] as well as PLA_2_ shows an anticoagulant effect [67], which makes exacerbates hemorrhage inside victims.

PLA_2_ are known proteins that work as neurotoxins in snake venom [68,69,70]. PLA_2_ have many neurotoxic properties including binding to the pre- or post-synaptic region, disrupting neuronal cell membrane potential, and even impairing neurotransmitter release and uptake. An experimental study showed that PLA_2_ destroys cholinergic and GABAergic cells in ganglion cell layers of the developing chick retina [71], suggesting the potential risk of vision loss, as a consequence of retinal ganglion cell (RGC) death. These neurotoxins might explain why ptosis and phobia are reported after snakebite. PLA_2_ is also one of the snake venom neurotoxins in *Bungarus multicinctus*, which causes ptosis, diplopia, and photophobia in patients following snakebite [72]. Pre-synaptic toxins (β-neurotoxins) inhibit acetylcholine (ACh) release from nerve terminals, which cause motor nerve disorders [73]. Post-synaptic toxins (α-neurotoxins) can strongly block nicotinic acetylcholine receptors (nAChR) [74], which leads to the blockade of the nerve impulse transmission [75]. Such toxic effects may cause pupillary constriction or synapse dysfunction further leading to photophobia or diplopia, respectively.

Ocular inflammation such as keratitis, iritis, and uveitis are common complications after snakebite. In snake venom, several enzymatic toxins including SVMPs, serine proteases, LAAOs, and PLA_2_ are known to be involved in immune modulation, all of which can cause inflammatory cytokine secretion by immune cells [55].

The mechanism of snakebite-induced glaucoma is still unclear. It might be a secondary ophthalmia of other ocular complications such as cataract, ocular ischemia, and uveitis, which are known to be risk factors for AACG. Rapid pupillary constriction is known to cause AACG. Thus, neurotoxin-induced rapid pupillary constriction could be another possible cause of AACG. In addition, AACG could be the result of coagulopathy-induced intraocular pressure (IOP) elevation. Although one study showed that the neurotoxins of snake venom cause RGC death [71], more investigations will be needed to verify the correlation between neurotoxin-induced RGC death and glaucoma.

## 4. Treatment

### 4.1. Treatment of Eye Injury Caused by Spitting Venom/Sprayed Toxin

Most patients suffering from snake venom-spit/toxin-spray usually irrigate their affected eyes either by tap water or saline before seeing a doctor. According to clinical observations, most of their ocular complications were alleviated or even disappeared (77%) a few days after immediate first-aid procedure [10]. Occasionally, venom of the spitting cobra in Nigeria (*N. nigricollis*) causes permanent blindness due to severe corneal damage [18]. It might be due to a high venom affinity to the cornea after exposure, which cannot be washed out even with immediate irrigation [51]. Topical corticosteroids, antibiotics, antihistamine, and anesthetic eye drops have been reported as treatments for snake venom-spit ophthalmia [3]. Since there is no evidence showing that the antivenom treatment alleviates venom-spit ophthalmia, topical or intravenous antivenom treatment is neither necessary nor recommended [3]. Heparin, owing to its chelating effect, has been shown to effectively reduce venom-induced ocular complication in experimental studies [49,76]; however, it is not yet approved for clinical use.

### 4.2. Novel Strategies for Treating Snake Envenoming

Intravenous administration of antivenom is the mainstay treatment for snakebites [77]. However, venom-induced ocular complications may occur despite the antivenom treatment (Table 2). In the eye, retinal hemorrhage is one of the most common complications and the lead cause of blindness following snakebite. SVMPs are the main toxins responsible for causing hemorrhage in animal skin models [61,62]. Thus, inhibition of SVMPs has been considered as a potential therapeutic strategy for snakebite-induced hemorrhage. Recently, a convincing metal chelator, 2,3-dimercapto-1-propanesulfonic acid (DMPS) was found to block the activity of Zn^2+^-dependent SVMPs in vitro [78]. In that study, they demonstrated that the 15 min-delayed treatment of DMPS using intraperitoneal (IP) injection prolonged the survival in venom envenoming in mouse models. In addition, oral administration of DMPS significantly enhanced the effect of antivenom treatment for preventing the animal from death [78]. The advantage of DMPS is its ability to neutralize SVMPs from different snake species. Therefore, DMPS could be a promising adjuvant treatment for SVMP-induced retinal hemorrhage. Although encouraging results are reported in a preclinical model, a proper clinical trial remains crucial for the development of DMPS as the first-aid medicine for snakebites, especially with respect to proper dosing and clinical guidelines [79].

Neurotoxins in venom can cause respiratory failure via paralysis of skeletal muscle [80] as well as cause AACG by rapid pupillary constriction or killing of RGCs [71]. Based on sequences from the most lethal elapid venoms worldwide, a synthetic recombinant peptide against the short-chain of α-neurotoxin (ScNtx) was developed [81]. In the animal study, the authors utilized ScNtx to generate the antivenom antibody by immunizing horses, and proved the efficacy of neutralizing α-neurotoxins and neurotoxic venoms from diverse genera such as *Micrurus*, *Dendroaspis*, *Naja,* and *Walterinnesia* [82]. These results provide a new strategy for developing next-generation antivenoms with higher effectiveness and broader neutralizing capacity.

## 5. Conclusions

Among the land snakes worldwide, spitting cobras (*Naja*) are the most common species to contribute to venom ophthalmia. Based on clinical reports (Table 1), they can spit as far as 2 m, which indicate a dangerous zone in a circle of 2-m diameter around the spitting cobra. Immediate eye irrigation is always the first procedure to remove venom from the eye, followed by topical antibiotics, corticosteroids, and analgesics. Anterior segment complications such as corneal injury, conjunctivitis, Keratitis, and blepharitis are usually diagnosed following venom ophthalmia, and most patients recovered without sequelae within 1–2 weeks. However, African cobras (*N. nigricollis*) can cause severe ocular complications leading to permanent blindness, suggesting that the venom of *N. nigricollis* seems more toxic to the eye. Venom ophthalmia caused by rattlesnakes (*C. atrox*) and tiger keelbacks (*R.t. tigrinus* and *R.t. formosanus*) are similar to spitting elapids in spite of different main toxic components. Overall, immediate ocular irrigation with generous volumes of tap water or saline is recommended for venom ophthalmia. Topical use with local anesthetic drops and use of vasoconstrictors such as epinephrine is advisable.

Regarding ocular complications caused by snake venom injection, interpreting the causal relationship and risk factors in these patients may be challenging due to scarce case reports and limited human studies. In this review, we noticed that the hemotoxin of vipers may cause severe posterior segment complications, secondary to anterior segment complications, despite administration of ample antivenom (Table 2). Impoverished regions lacking health resources may experience delay in administering antivenom or insufficient supportive treatment, which partly contribute to the increased prevalence of ocular injury and permanent blindness. A case series reported that a pit viper (*Protobothrops mucrosquamatus*) bite to the head did not show any ocular complications [83], suggesting that a site of injury close to the eyes may not be associated with snake venom injection-induced ocular complications. It is unclear if prompt antivenom administration might be the key factor for preventing ocular complications after snake venom injection. Currently, administration of intravenous antivenom, as soon as possible, is likely to be the most effective treatment though novel therapeutic development strategies for neutralizing toxins of snake venom, such as biosynthetic oligoclonal antivenom (BOA) [84], ScNtx [82], and DMPS [78] have shown promising results in a preclinical model. 

## Figures and Tables

**Table 1:** Venom ophthalmia caused by venomous snakes.

Species	Family	Gland	Venom Components	Spitting Distance (Meter)	Ocular Lesions	Treatments	Outcome	Ref.
*Naja atra*	*Elapidae*	Duvernoy	α-Neurotoxin PLA_2_ LAAO CTXs	0.7–1	Conjunctivitis; corneal injury; Keratoconjunctivitis; Keratitis; Photophobia	Water irrigations; topical antibiotics, corticosteroids, and vasoconstrictors; Analgesics	Full recovery	[3,9,10]
*N. naja*	*Elapidae*	Duvernoy	α-Neurotoxin PLA_2_ LAAO CTXs	0.2	conjunctival inflammation and swelling of the eyelids	Water irrigations	Full recovery	[3]
*N. nigricollis*	*Elapidae*	Duvernoy	α-Neurotoxin PLA_2_ LAAO CTXs	0.8–2	Corneal injury; hypopyon; uveitis; conjunctival hyperemia	Water irrigations; topical antibiotics, corticosteroids atropine; Analgesics	Recovered with sequelae; Few blind	[3,11,16,17,18]
*N. pallida*	*Elapidae*	Duvernoy	α-Neurotoxin PLA_2_ LAAO CTXs	0.5–1	Blepharitis, blepharospasm, conjunctivitis	Water irrigations	Full recovery	[3]
*N. siamensis*	*Elapidae*	Duvernoy	α-Neurotoxin PLA_2_ LAAO CTXs	2	Conjunctivitis	Water irrigations; Epinephrine	Full recovery	[3]
*Hemachatus haemachatus*	*Elapidae*	Duvernoy	PLA_2_ LAAO CTXs	1	Red eye	Water irrigations	Full recovery	[3]
*Crotalus atrox*	*Viperidae*	Duvernoy	PLA_2_ LAAO SVMPs	n/a	Photophobia	Water irrigations; Topical antivenom, Analgesics	Full recovery	[12,13,14]
*Rhabdophis tigrinus*	*Colubridae*	Nuchal	bufadienolides	n/a	Conjunctivitis, keratitis, corneal clouding	Saline irrigations; topical antibiotics, corticosteroids	Full recovery	[24,25,26]
*R.t. formosanus*	*Colubridae*	Nuchal	bufadienolides	1	Keratitis, corneal stromal edema and conjunctival congestion	Water irrigation; topical corticosteroid, antihistamine and antibiotic	Full recovery	[27]

PLA_2_: Phospholipases A_2_; LAAO: L-amino acid oxidases; CTXs: Cardiotoxins; SVMPs: Snake venom metalloproteinases.

**Table 2:** Ocular complications following snake venom injection.

	Species	Sex/Age	Other Symptoms	Presuming Venom Components	* Possible Direct Mechanism ^#^ Possible Indirect Mechanism	Antivenom/ Amount	Outcome	Ref.
Anterior segment manifestations								
Subconjunctival hemorrhage	Unknown	M/70	Acute renal failure	SVMPs	* Proteolytic effect	IV Antivenom/ 16 vials	Recovery	[35]
Corneal striae, anterior pseudohypopyon, cataract	Unknown	M/70	Acute renal failure	Hemotoxin	^#^ Secondary of subconjunctival hemorrhage and side effect of antivenom	IV Antivenom/ 16 vials	Remain corneal striae and cataract	[35]
Cataract	Unknown	M/60	AMS	SVMPs	^#^ Anterior inflammation caused by vitreous hemorrhage	IV Antivenom/ Unclear	NLP	[36]
Iris atrophy, Cataract	Unknown	M/55	Anasarca	Cytotoxin	* Anterior ischemia	IV Antivenom/ Unclear	IMC	[36]
Iris atrophy, Cataract	Unknown	M/45	Limb edema	Cytotoxin	* Anterior ischemia	IV Antivenom/ Unclear	Lost for follow-up	[36]
Posterior segment manifestations								
CRAO	Unknown	M/26	Headache	Hemotoxin	* Coagulopathy	Unclear	Reduction of vision	[32]
CRAO	Unknown	M/24	Bleeding diathesis	Hemotoxin	* Coagulopathy	As above	Reduction of vision	[32]
CRAO	Viper	F/30	AMS	Hemotoxin,	* Coagulopathy	IV Antivenom/ Unclear	Poor visual prognosis	[30]
CRAO	Viper	F/17	n/a	Hemotoxin	* Coagulopathy	IV Antivenom/ Unclear	Lost for follow-up	[29]
Retinal or vitreous hemorrhage	Viper	F/17	AMS	SVMPs	* Proteolytic effect	IV Antivenom/ Unclear	NLP	[33]
Vitreous hemorrhage	Viper	M/60	AMS	SVMPs	* Proteolytic effect	IV Antivenom/ Unclear	20/200 for O.U.	[36]
Macular infarction	Viper	F/17	AMS	Hemotoxin	* Thrombosis	IV Antivenom/ Unclear	NLP	[33]
Retinal detachment	Unknown	F/13	Vitreous hemorrhage	SVMPs	* Hemorrhage-induced subretinal edema	IV Antivenom/ Multiple dose	20/200 for O.S. Reduction of vision for O.D.	[34]
Glaucoma								
AACG	Unknown	M/70	Acute renal failure	Hemotoxin Neurotoxin	^#^ Capillary leak due to hematoxin * Ophthalmoplegia due to neurotoxin	IV Antivenom/ 16 vials	Lost for follow-up	[35]
AACG	*Vipera lebetina*	F/67	AMS	Hemotoxin, Neurotoxin	* Coagulation cascade and synapse disorder	IV Antivenom/ Unclear	BCVA is 5/10 for O.S.	[39]

AMS: Altered mental status; SVMPs: Snake venom metalloproteinases; NLP: No light perception; IMC: Immature cataract; BCVA: Best corrected visual acuity.
